# Menopausal Symptoms among Postmenopausal Women of a Selected Municipality: A Cross-sectional Survey

**DOI:** 10.31729/jnma.7052

**Published:** 2021-11-30

**Authors:** Sitasma Sharma, Laxmi Adhikari, Isha Karmacharya, Maheshor Kaphle

**Affiliations:** 1 Nepal Health Research Council, Ramshah Path, Kathmandu, Nepal; 2 Department of Public Health, Central Institute of Science and Technology, Kathmandu, Nepal

**Keywords:** *menopause*, *middle aged*, *Nepal*, *postmenopausal*

## Abstract

**Introduction:**

Postmenopausal women experience various menopause-specific somatic, psychological, and urogenital symptoms which tend to affect their overall well-being. However, there is a scant focus on menopausal health problems of postmenopausal women in Nepal. The aim of the study was to find out the prevalence of menopausal symptoms among postmenopausal women of a Municipality.

**Methods:**

A cross-sectional survey was conducted in selected wards of Tokha Municipality of Kathmandu district among postmenopausal women aged 45-60 years from September to October 2019. Ethical approval was taken from the Ethical Review Board, Nepal Health Research Council (reference number 694). Random sampling was used as the sampling technique. Face-to-face interview was used for data collection while a semi-structured interview schedule including the Nepali version of Menopause Rating Scale was used as a tool for measuring menopausal symptoms. EpiData version 3.1 was used for data entry while data analysis was performed using Statistical Packages for the Social Sciences version 20.

**Results:**

Amongst the postmenopausal women, all 203 (100%) had menopausal symptoms with majority reporting milder symptoms as found in 143 (70.4%). The mean Menopause Rating Scale (MRS) score was obtained as 13.21±5.1. The most prevalent moderate and mild symptoms were vaginal dryness 94 (46.3%), and depressive mood 71 (35%) respectively while physical and mental exhaustion 175 (86.2%) was the most common in all forms. Over half 102 (50.2%) of the respondents were unaware of menopausal symptoms and almost three-fifth 121 (59.6%) reported menopause related symptoms affected their daily work activities.

**Conclusions:**

The majority of postmenopausal women in this study had milder menopausal symptoms, which parallels findings from other national studies.

## INTRODUCTION

Post-menopause phase is the stage after the occurrence of menopause where women experience amenorrhea for a year or more.^1^ With the growing pattern of global life expectancy, it is projected that proportion of perimenopausal or postmenopausal women living in developing countries will grow up to 76%.^2^ Some studies suggested that menopausal symptoms are more prevalent in postmenopausal women than in premenopausal and perimenopausal women.^3^

In Nepal, different studies reported high prevalence of menopausal symptoms among postmenopausal women indicating undesirable effect on their physical, psychological, sexual, and social health thus on their overall quality of life.^4,5^ Existing evidence indicate inadequate awareness including scant focus on menopausal health problems of women of late reproductive age and postmenopausal status in Nepal.^6^'^7^

This study has attempted to find out the menopausal symptoms among postmenopausal women in Tokha Municipality of Kathmandu.

## METHODS

A cross-sectional survey was conducted among postmenopausal women aged 45-60 years in selected wards of Tokha Municipality, Kathmandu. The study duration was from May to November 2019. Permission was obtained from the Tokha Municipality Office and ethical approval was taken from Ethical Review Board of Nepal Health Research Council (reference number 694) prior to conducting the study. Data were collected between 18 September to 20 October. Written informed consent from the respondents was obtained prior to data collection and those who had used hormone replacement therapy in the previous six months, had a history of oophorectomy and/or hysterectomy, physical and mental disability, and had refused to give consent were excluded. Anonymity and confidentiality of the respondents were maintained ensuring their voluntary participation.

Two-stage random sampling was done where the first stage was cluster sampling, and the second stage was Probability Proportional to Size (PPS) method. The 11 wards comprising Tokha Municipality were taken as the total clusters for the study. After calculating the cumulative female population and determining the sampling interval as 760, the first study cluster was selected as ward number three of Tokha Municipality. Accordingly, a total of seven wards were selected as the study clusters. The PPS method was applied for determining the number of samples to be collected from each randomly selected cluster. At first, the center of each selected cluster/ward was identified using the map provided by the municipality office. The direction to choose the starting household in each cluster was determined by using the Spin the Bottle method where all the households were counted along that direction until the boundary was reached. Then a random number from 1 through the 'x' number of households counted was chosen, which denoted the first household for data collection.^8^

As per National Population and Housing Census 2011 of Nepal,^9^ women aged 45-59 years comprised 11.01% of the total female population. Taking this national proportion as a reference, the female population of the age group 45-59 years at Tokha was calculated as 5320 and was taken as the total population of females in Tokha.

The sample size was estimated using the formula,

n_o_ = Z^2^ × p × (1-p) / e^2^

  = (1.96)^2^ × (0.86) × (1-0.86) / (0.05)^2^

  = 185.011

Where,

n_o_ = minimum required sample size
Z= 1.96 at 95% Confidence Interval (CI)
p= past prevalence of menopausal symptoms among postmenopausal women taken from a previous study, 86%^10^
q= 1-p
e= margin of error, 5%

Now, adjusting the sample size for finite population,

n = N × n_o_ / (n_o_ + N - 1)

  = (5320 × 185.011)/(185.011+5320-1)

  = 178.82

Where,

n= adjusted sample size
N= total population of females aged 45-59 years at Tokha municipality^9^

Therefore, the calculated sample size was 179. Adding a 10% non-response rate, the sample size of 197 was reached. However, we collected data from 203 women.

Data collection was carried out from September to October 2019 whereas the data collection technique applied was face to face interview. Interview schedule was used as a study tool which consisted of semistructured researcher designed questionnaire and standard questionnaire i.e. Menopause Rating Scale (MRS). Menopause Rating Scale (MRS), validated in Nepal, was used as a standard tool for the study after obtaining the author's permission.^10^ It is an internationally valid health-related quality of life and screening tool and was developed in the 1990s in Germany.^11^ It consists of three subscales - somato-vegetative scale, psychological scale, and urogenital scale-consisting of eleven items for the assessment of menopausal symptoms. Each item of the MRS scale has five ordinal scales ranging from zero to four for rating menopausal symptom where zero indicates no symptom and four indicates very severe symptom. The somato-vegetative domain consists of four items-hot flushes, heart discomfort, sleep problems, and joint and muscular discomfort. The psychological domain comprises four items-depressive mood, irritability, anxiety, and physical and mental exhaustion. The urogenital domain includes three items-sexual problems, bladder problems, and dryness of the vagina. The sum of item scores in a particular subscale is the overall score for that subscale. The overall MRS score ranges from 0 to 44. For the study purpose, postmenopausal women were categorized as respondents with mild menopausal symptoms (MRS cut-off score <16) and with severe menopausal symptoms (MRS cut-off score ≥16), as determined by a previous study among Nepalese middle-aged women and the same was taken in this study.^10^ Those respondents reporting a higher number of symptoms along with the higher score listed in the MRS were suggested to consult a physician or gynecologist at the end of the data collection process.

For the analysis purpose, the data entered in Epi-Data version 3.1 was transferred to Statistical Packages for the Social Sciences (SPSS) version 20. Variables were re-coded and transformed before the final analysis. Descriptive frequency, percentage, mean, and standard deviation were calculated to summarize the sociodemographic characteristics, prevalence and severity of menopausal symptoms, age of menopause, mean MRS score, knowledge related variables, and social life related variables.

## RESULTS

Amongst 203 respondents, the mean age of menopause was found to be 48.35±4.54 years with a minimum of 35 and a maximum of 58. About 162 (79.8%) of the respondents were more than 50 years of age. The most 143 (70.4%) of the respondents were housewives involved in household work followed by businesswomen at 36 (17.7%). Almost half 99 (48.8%) of the respondents were illiterate. The majority 129 (63.5%) of the respondents belonged to the privileged group. Likewise, most of the respondents were married 172 (84.7%) (Table 2). More than half 102 (50.2%) of the respondents were not aware of menopausal symptoms. About 121 (59.6%) of the postmenopausal women said that menopausal related symptoms affected their daily work activities.

The mean Menopause Rating Scale (MRS) score was obtained as 13.21 ±5.1. Categorically, symptoms of psychological subscale 138 (67.9%) in all forms (mild, moderate and severe) were more prevalent than symptoms of urogenital and somatic subscale. Among the psychological subscale, the commonly prevailing menopausal symptom was physical and mental exhaustion 175 (86.2%). Similarly, joint and muscular discomfort 158 (77.8%) was the most prevalent symptom on the somatic subscale, while vaginal dryness 174 (85.7%) was the most common symptom on the urogenital subscale (Table 1).

**Table 1:** Prevalence of menopausal symptoms in all forms perceived by respondents.

Menopausal Symptoms	All forms^*^ n^†^ (%)	Mean±SD
Somatic subscale	123 (60.35)	4.73±2.6
Hot flushes	131 (64.6)	1.37±1.3
Heart discomfort	81 (40.9)	0. 64±0.9
Sleep problems	118 (58.1)	1. 02±1.1
Joint and muscular discomfort	158 (77.8)	1.70±1.1
Psychological subscale	138 (67.87)	4.72±2.8
Depressive mood	120 (59.1)	0.91±0.9
Irritability	132 (65.1)	1.12±1.1
Anxiety	124 (61.1)	0.95±0.9
Physical and mental exhaustion	175 (86.2)	1.75±1.1
Urogenital subscale	134 (66)	3. 76±1.8
Sexual problems	154 (75.9)	1.76±1.2
Bladder problems	74 (36.4)	0.60±0.9
Dryness of vagina	174 (85.7)	1.50±0.87

* All forms include mild, moderate, severe, very severe,

† Multiple responses

**Figure 1:**
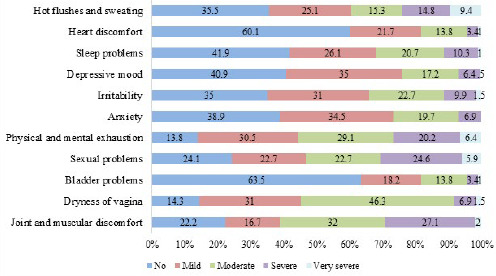
Perceived severity of menopausal symptoms in the Menopause Rating Scale.

All postmenopausal women included in this study reported experiencing at least one menopausal symptom listed in the Menopause Rating Scale. Most of the symptoms reported were in the mild and moderate forms. In the moderate form, the most prevalent symptoms were vaginal dryness 94 (46.3%), followed by joint and muscular discomfort 65 (32%), and in the mild form, depressive mood 71 (35%), anxiety 70 (34.5%), and physical and mental exhaustion 62 (30.5%). Joint and muscular discomfort was the most prevalent severe symptom reported by respondents 55 (27.1%), followed by sexual problems 50 (24.6%), and physical and mental exhaustion 41 (20.2%). Likewise, the very severe symptom reported was hot flushes and sweating 19 (9.4%) followed by physical and mental exhaustion 13 (6.4%) (Figure 1).

The majority 143 (70.4%) of the respondents had Menopause Rating Scale score of less than 16, indicating greater prevalence of milder form of menopausal symptoms (Table 2).

**Table 2:** Socio-demographic characteristics and severity of menopausal symptoms among the respondents.

Socio-demographic Characters		Severity of Menopausal Symptoms		
		Mild Symptoms (MRS <16) (n=143) n (%)	Severe Symptoms (MRS ≥ 16) (n=60) n (%)	Total (n = 203) n (%)
Age Group	<=50 years	32 (78)	9 (22)	41 (20.2)
	>50 years	111 (68.5)	51 (31.5)	162 (79.8)
Occupation	Non-working	98 (68.5)	45 (31.5)	143 (70.4)
	Working	45 (75)	15 (25)	60 (29.6)
Education	Literate	76 (73.1)	28 (26.9)	104 (51.2)
	Illiterate	67 (67.7)	32 (32.3)	99 (48.8)
Ethnicity	Privileged	91(70.5)	38 (29.5)	129 (63.5)
	Underprivileged	52 (70.3)	22 (29.7)	74 (36.5)
Marital status	Married	124 (72.1)	48 (27.9)	172 (84.7)
	Currently single	19 (61.3)	12 (38.7)	31 (15.3)

## DISCUSSION

This study found the mean age of menopause to be 48.35 years. This finding is consistent with previous studies carried out in Nepal.^6-10^ The findings from other countries like India, Pakistan and Sri Lanka showed a similar range for age of menopause in their studies.^12-14^ In the present study, more than one-fourth (29.6%) of the postmenopausal women had MRS score greater than 16, which is comparable with a previous study carried out in Nepal where 23% of the postmenopausal women had MRS score greater than 16.

Postmenopausal women of this study reported different menopausal symptoms listed in Menopause Rating Scale among which the most prevalent menopausal symptom in mild form was depressive mood (35%). This result is comparable with a previous study done in Nepal, where the most commonly reported mild form of menopausal symptom also was depressive mood (52.4%)^15^ although the reported percentage is higher, which may be due to differences in the study settings and study population. In this study, the most prevalent menopausal symptoms (in all forms) were reported as physical and mental exhaustion (86.2%), dryness of vagina (85.7%) followed by joint and muscular discomfort (77.8%). These results are in accordance with a previous study done in Nepal, where most prevalent menopausal symptoms were physical and mental exhaustion (86%), joint and muscular discomfort (86%) followed by dryness of vagina (85%).^10^ In comparison, studies Lanka from Sri^3^. Malaysia,^16^ and Saudi Arabia^17^ have found physical and mental exhaustion (53%), joint and muscular discomfort (73.3%), and joint and muscle pain (80.7%) to be the most prevalent menopausal symptoms in postmenopausal women respectively.

Overall, this study found symptoms of psychological subscale (67.87%) to be more prevalent followed by symptoms of urogenital and somatic subscales, which is consistent with the findings obtained from a study carried out in India. However, the prevalence of psychological problems (78%) in the previous study is higher than in the present study which may be attributed to differences in study settings as the previous study was carried out in health care centers in India.^13^ Similarly, a community-based study from Pakistan also found psychological symptoms to be the most prevalent postmenopausal symptoms.^14^

In contrast, a study from Kavre, Nepal reported vasomotor symptoms to be highly prevalent (68%) among all the menopausal symptoms in postmenopausal women.^5^ This dissimilarity in the findings may be due to the difference in the use of study tools and differences in socio-cultural and economic context as the respondents were mostly from the marginalized group. Another hospital-based study from Nepal showed physical symptoms to be the most common symptoms reported by the perimenopausal and postmenopausal women.^18^ Likewise, prior studies from the countries like India^19^ and Egypt^20^ have found physical symptoms to be highly prevalent in comparison to other symptoms. In addition, a systematic review study carried out among Asian middle-aged women also found physical symptoms to be most prevalent followed by psychological and somatic symptoms.^21^ In general, the differences in socio-cultural background, methodological procedure such as use of study tools, and the study designs applied while conducting research may be attributed to variations in reported prevalence of menopausal symptoms across the countries and regions.

In the present study, more than half (50.2%) of the respondents did not have knowledge on menopausal symptoms which reflects the result obtained from previous studies conducted in Nepal.^6,22^ The possible explanation for this might be that Nepalese women accept menopausal phenomena as a normal situation of life due to which they do not associate it with medical conditions capable of posing threats to their health condition.^6^ On the other hand, 59.6% of women reported menopausal symptoms affected their daily work performance to some extent which is comparable to the prior study from Nepal reporting 46% of the women experiencing difficulties in their daily activities due to these symptoms.^23^

The transition through menopause is a life event that can profoundly affect overall well-being and quality of life of menopausal women.^3-5^ Previous studies from Nepal and Sri Lanka have reported that menopause-specific symptoms highly diminish the overall wellbeing and quality of life of postmenopausal women in comparison to premenopausal and perimenopausal women.^3,4^ Although many middle-aged women in Nepal as well as the respondents of this study perceived menopause as a natural process, they had inadequate awareness and knowledge regarding menopausal phenomena, menopausal symptoms, and how these changes might have been affecting their overall wellbeing and quality of life.^4^ As two largest fractions of Nepalese population will be comprised of 45-49 and 50-54 age groups by the year 2050,^24^ Nepalese women will spend a longer time in postmenopausal phase indicating the need for increased attention to the health of women belonging to postmenopausal category.

The present study has some limitations. When reporting and assessing the severity of menopausal symptoms there could have been an element of subjectiveness as well as respondents may have had recall bias while reporting the exact duration of menopause. In addition, another limitation of this study was confounding variables such as presence of health problems like hypertension, thyroid problems, and diabetes were not excluded from the study, which sometimes are similar to the menopausal symptoms. Thus, the results of the present study should be carefully viewed in the light of research limitations mentioned above.

## CONCLUSIONS

The postmenopausal women in this study most commonly experienced milder menopausal symptoms, which is similar with findings from other national studies. On the other hand, most postmenopausal women were not aware of menopausal symptoms in this study. So, adequate assessment of health problems along with health needs of postmenopausal women and conduction of menopause specific awareness activities at local levels are of utmost importance to enhance the general well-being of these group of women.
